# Sustainable Tumor-Suppressive Effect of iPSC-Derived Rejuvenated T Cells Targeting Cervical Cancers

**DOI:** 10.1016/j.ymthe.2020.07.004

**Published:** 2020-07-09

**Authors:** Tadahiro Honda, Miki Ando, Jun Ando, Midori Ishii, Yumi Sakiyama, Kazuo Ohara, Tokuko Toyota, Manami Ohtaka, Ayako Masuda, Yasuhisa Terao, Mahito Nakanishi, Hiromitsu Nakauchi, Norio Komatsu

**Affiliations:** 1 Department of Hematology, Juntendo University School of Medicine, 2-1-1 Hongo, Bunkyo-ku, Tokyo 113-8421, Japan; 2 Division of Stem Cell Therapy, Distinguished Professor Unit, The Institute of Medical Science, The University of Tokyo, 4-6-1 Shirokanedai, Minato-ku, Tokyo 108-8639, Japan; 3 TOKIWA-Bio, Inc., Tsukuba Center Inc. (TCI), Building G, 2-1-6 Sengen, Tsukuba, Ibaraki 305-0047, Japan; 4 Department of Obstetrics and Gynecology, Juntendo University School of Medicine, 2-1-1 Hongo, Bunkyo-ku, Tokyo 113-8421, Japan; 5 National Institute of Advanced Industrial Science and Technology (AIST), Tsukuba, Ibaraki 305-8565, Japan; 6 Institute for Stem Cell Biology and Regenerative Medicine, Stanford University School of Medicine, 265 Campus Drive, Stanford, CA 94305, USA

**Keywords:** safer iPSCs, HPV-CTL, human papilloma virus type 16, cervical cancer, rejuvenated CTL

## Abstract

Immunotherapy utilizing induced pluripotent stem cell (iPSC) technology has great potential. Functionally rejuvenated cytotoxic T lymphocytes (CTLs) can survive long-term as young memory T cells *in vivo*, with continuous tumor eradication. Banking of iPSCs as an unlimited “off-the-shelf” source of therapeutic T cells may be feasible. To generate safer iPSCs, we reprogrammed human papilloma virus type 16 (HPV16) E6-specific CTLs by Sendai virus vector without cotransduction of SV40 large T antigen. The iPSCs efficiently differentiated into HPV16-specific rejuvenated CTLs that demonstrated robust cytotoxicity against cervical cancer. The tumor-suppressive effect of rejuvenated CTLs was stronger and more persistent than that of original peripheral blood CTLs. These rejuvenated HPV16-specific CTLs provide a sustained tumor-suppressive effect even for epithelial cancers and constitute promising immunotherapy for cervical cancer.

## Graphical Abstract

**Figure:**
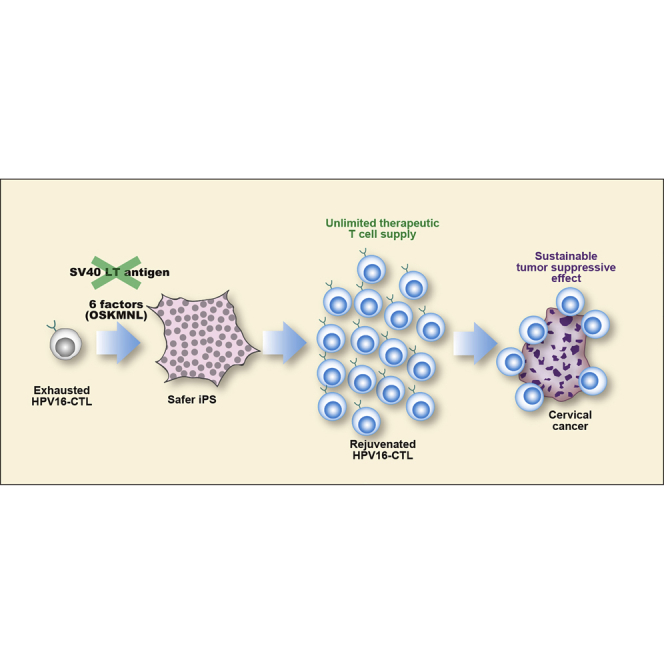

## Teaser

In this article, Honda and colleagues show that safer iPSCs can be established from an HPV-CTL clone by Sendai virus vector without cotransduction of SV40 large T antigen. The iPSCs efficiently differentiated rejuvenated CTLs and exhibited a sustained cervical cancer-suppressive effect *in vitro* and *in vivo*.

## Introduction

Human papilloma virus (HPV) causes oropharyngeal cancer, cervical cancer, and other genital cancers. There are at least 170 HPV genotypes, classified as high-risk types and low-risk types. HPV16 and HPV18 are the most prevalent high-risk types related to cervical cancer, causing more than 70% of cases.^1^, ^2^, ^3^, ^4^, ^5^ Prophylactic HPV vaccines are effective in prevention of HPV infection but not in treating established cancers.^6^^,^^7^ Cervical conization for patients with HPV-related epithelial dysplasia increases future risk of premature delivery;^8^ total hysterectomy for early-stage cancers of course abolishes fertility. Metastatic cervical cancer is highly refractory to chemotherapy and its prognosis is extremely poor.^9^^,^^10^ Therefore, development of cervical cancer treatment should be a top priority.

Adoptive T cell therapy can induce durable remission in some tumors (e.g., melanoma^11^). As E6 and E7 are key oncoproteins in cervical cancer, contributory to tumorigenesis, constitutively expressed by cervical cancer cells, and absent from healthy tissues, they are ideal targets of T cell therapy.^6^^,^^12^, ^13^, ^14^ However, adoptive T cell therapy against solid cancers is challenging, because solid cancers exhibit reduced susceptibility to T cell-mediated destruction in the tumor microenvironment.^15^ Moreover, T cells often become exhausted when continuously exposed to their target antigens,^16^ although a sustained anti-cervical cancer effect is needed.

Induced pluripotent stem cell (iPSC) technology augments cytotoxicity of virus-specific cytotoxic T lymphocytes (CTLs).^17^^,^^18^ Virus-specific CTLs generated from T cell-derived iPSCs (T-iPSCs) have higher proliferative capacity and longer telomeres than do original peripheral blood-derived CTLs, which means that iPSC-derived CTLs are functionally rejuvenated (rejuvenated T cells [rejTs]).^17^, ^18^, ^19^ Epstein-Barr virus (EBV)-specific rejTs persist as central memory T cells *in vivo* for at least 6 months, resulting in robust and continuous eradication of refractory EBV-associated lymphoma.^20^ T-iPSCs as an unlimited source of rejuvenated CTLs offer promise for cervical cancer treatment.

We asked whether virus-specific rejTs directed to HPV16 E6 and E7 are effective in cervical cancer treatment. T-iPSCs established from HPV16 E6- or E7-specific CTLs efficiently differentiated into HPV16-specific rejTs that exhibited robust and sustained cytotoxicity against cervical cancer.

## Results

### HPV16 E6-Specific CTLs and E7-Specific CTLs Were Generated from Healthy Donors

**Figure 1:**
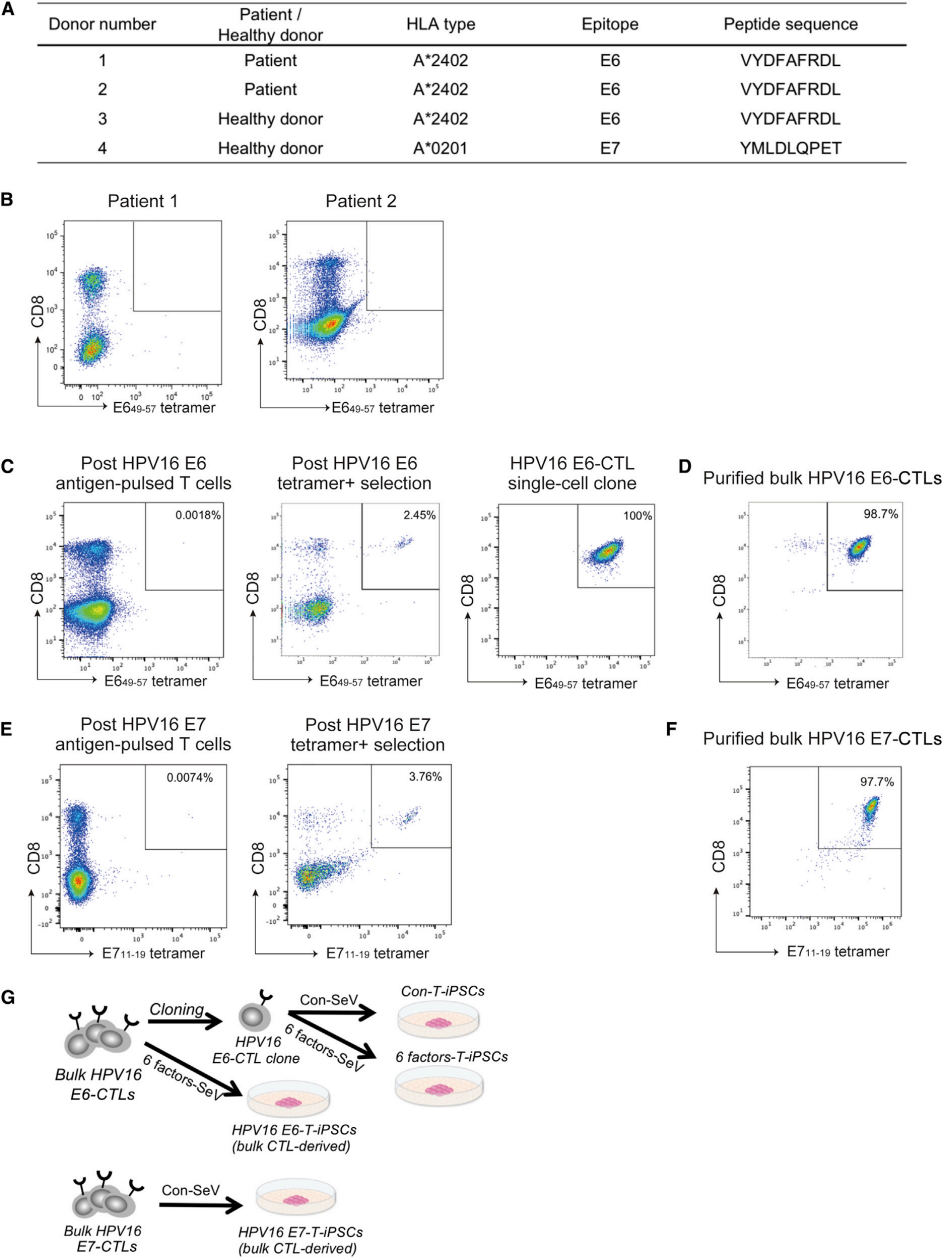
Generation of HPV16 E6- and E7-Specific CTLs (A) Donor characteristics and CTL epitope. (B) Flow cytometric E6_49–57_ tetramer analysis of two patient donors’ lymphocytes 7 days after peptide pulse. (C) Flow cytometric E6_49–57_ tetramer analysis of a healthy donor’s lymphocytes 7 days after peptide pulse (left). E6_49–57_ tetramer^+^ cells were bulk cultured (center) and single-cell cloned (right). (D) E6_49–57_ tetramer^+^ bulk-cultured CTLs were purified twice by fluorescence-activated cell sorting (FACS). (E) Flow cytometric E7_11–19_ tetramer analysis of a healthy donor’s lymphocytes 7 days after peptide pulse (left). E7_11–19_ tetramer^+^ cells were bulk cultured (right). (F) E7_11-19_ tetramer^+^ bulk-cultured CTLs were purified twice by FACS. (G) Schematic illustration of establishment of T-iPSCs from HPV16 E6- and E7-CTLs.

All donors were confirmed to express human leukocyte antigen (HLA)-A^∗^2402 or A^∗^0201 antigens (Figure 1A). We first tried to generate HLA-A^∗^2402-restricted HPV16 E6-specific CTLs from two patient donors with progressing disease and from a healthy donor. However, an HPV16 E6 tetramer^+^ population was not detected in the E6 peptide-pulsed cells; indeed, we failed in generating HPV16 E6-specific CTLs from the two patient donors (Figure 1B). Alternatively, HPV16 E6-specific CTLs strongly reactive against E6_49–57_ antigen were detected in 0.0018% of E6 peptide-pulsed T cells generated from the healthy donor (no. 3, Figure 1C, left). After tetramer^+^ cell selection, the proportion of tetramer^+^ cells rose to 2.46% (Figure 1C, center). We subsequently established an HPV-16 E6-specific CTL single-cell clone (Figure 1C, right). As single-cell cloning is time-consuming, we repeatedly sorted tetramer^+^ cells and also generated HPV16 E6-specific bulk CTLs that showed 98.7% antigen specificity (Figure 1D). Similarly, HPV16 E7-specific CTLs were successfully generated from an HLA-A∗0201^+^ healthy donor (no. 4, Figure 1A), constituting 0.007% of E7_11–19_ tetramer^+^ T cells (Figure 1E, left). E7 tetramer^+^ cells were repeatedly sorted (Figure 1E, right) to generate HPV16 E7-specific bulk CTLs. These showed 97.7% E7 antigen specificity (Figure 1F).

### HPV16 E6-Specific CTLs Could Be Reprogrammed into T-iPSCs without Cotransduction of SV40 Large T Antigen

We next reprogrammed an HPV16 E6-specific CTL clone that we had generated T-iPSCs. The clone was transduced with Sendai virus (SeV) vector and T-iPSCs were established. We have never succeeded in the establishment of T-iPSCs from a CTL clone by transduction solely of the four Yamanaka factors (OCT3/4, SOX2, KLF4, and c-MYC [OSKM]); cotransduction of SV40 large T antigen has been indispensable. However, use of SV40 large T antigen for reprogramming might increase double-strand break-associated mutation. Thus, for clinical use we attempted to establish safer T-iPSCs without cotransduction of SV40 large T antigen. We succeeded using 6 factors-SeV, which loads OSKM (four factors), NANOG, and LIN28 (six factors in all). We also transduced purified bulk HPV16 E6-CTLs (Figure 1D) with 6 factors-SeV in the same manner and could establish T-iPSCs. With respect to purified bulk HPV16 E7-CTLs (Figure 1F), we transduced cells with two SeV vectors (OSKM and SV40 large T antigen, conventional-SeV [Con-SeV]) or 6 factors-SeV, but only T-iPSCs transduced with Con-SeV could be established. In all, we established four T-iPSC lines: two from an HPV16 E6-CTL clone and the two bulk lines HPV16 E6-CTLs and HPV16 E7-CTLs (Figure 1G).

### SeV Vector Was Efficiently Cleared in T-iPSCs Reprogrammed by 6 Factors-SeV

**Figure 2:**
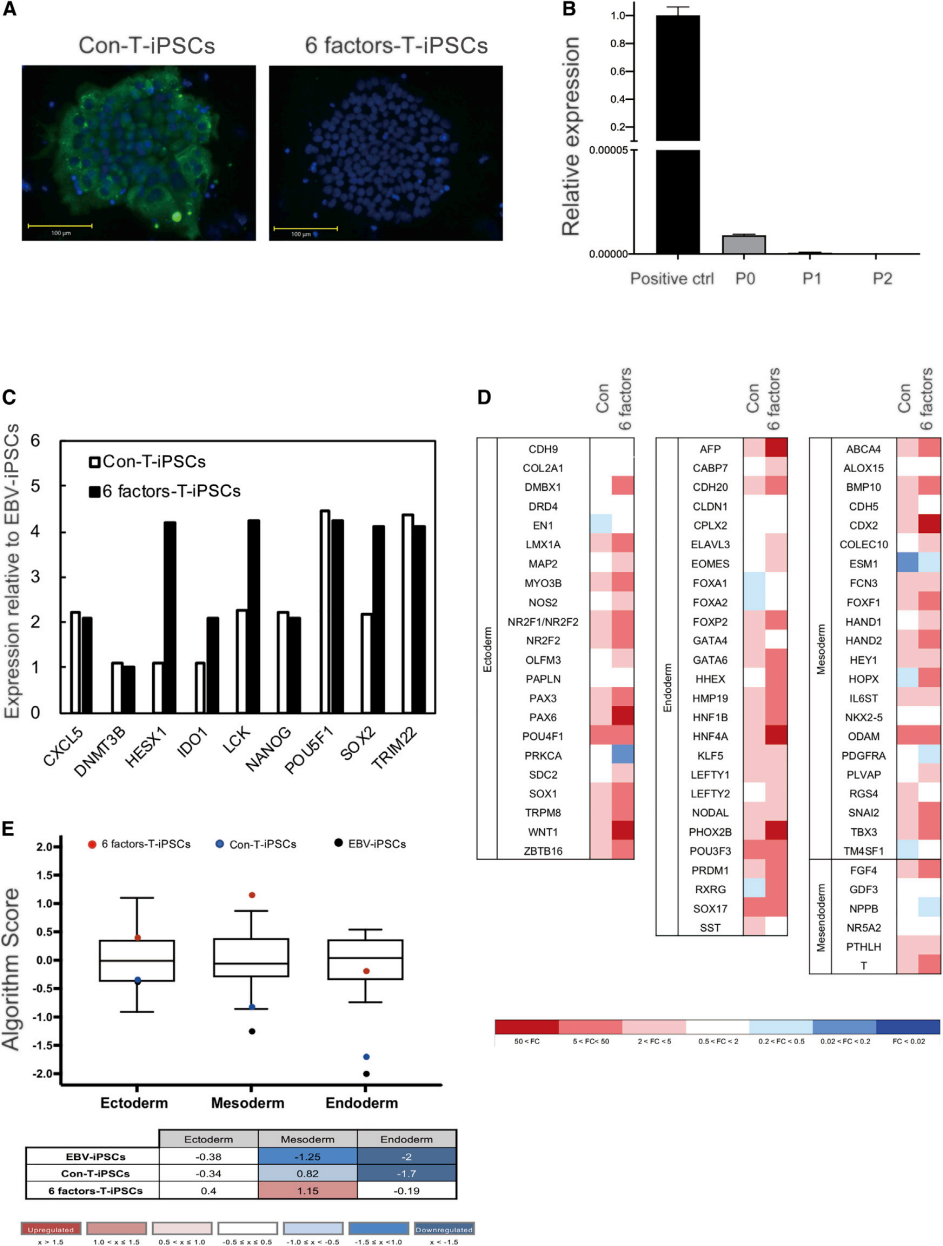
T-iPSC Establishment by Reprogramming with Con-SeV and 6 Factors-SeV (A) T-iPSCs were incubated with a primary antibody against SeV NP antigen (green). Cells were counterstained with DAPI (blue). Scale bars, 100 μm. (B) Quantitative real-time PCR analysis to evaluate residual 6 factors-SeV vector. Error bars represent ±SD. (C) Relative gene expression associated with self-renewal of Con-T-iPSCs and 6 factors-T-iPSCs was measured by a TaqMan hPSC Scorecard. EBV-iPSCs, control. (D) Relative expression of trilineage differentiation-associated genes in Con-T-iPSCs and 6 factors-T-iPSCs. EBV-iPSCs, control. (E) Trilineage differentiation-associated gene expression in Con-T-iPSCs, 6 factors-T-iPSCs, and EBV-iPSCs (all are undifferentiated) was analyzed by Scorecard cloud-based software (Thermo Fisher Scientific), with results graphed as a boxplot.

To examine SeV clearance in two T-iPSC lines reprogrammed with Con-SeV and 6 factors-SeV derived from HPV16 E6-CTL clone, these two T-iPSC lines in passage 2 were stained with anti-SeV nucleocapsid protein (NP) antibody and examined by fluorescence microscopy. Colonies of T-iPSCs reprogrammed with Con-SeV (Con-T-iPSCs) expressed SeV NP antigen, whereas established colonies of T-iPSCs reprogrammed with 6 factors-SeV (6 factors-T-iPSCs) did not express SeV NP antigen in passage 2 (Figure 2A). To measure residual SeV quantitatively, we performed quantitative real-time PCR. Relative expression of 6 factors-SeV against a positive control was detected in passage 0 (0.0000089 against 1) and in passage 1 (0.0000006 against 1) by quantitative real-time PCR, and complete clearance was confirmed by passage 2 (Figure 2B).

### T-iPSCs Reprogrammed by 6 Factors-SeV Efficiently Differentiated into HPV16 E6-rejTs

To characterize both T-iPSC lines established from HPV16 E6-CTLs, expression of genes associated with self-renewal and differentiation potential was analyzed using the TaqMan human PSC (hPSC) Scorecard panel. Expression of self-renewal genes relative to that in the control T-iPSCs established from EBV-specific CTLs (EBV-iPSCs) was at almost the same level in 6 factors-T-iPSCs as in Con-T-iPSCs or higher (Figure 2C). In measuring expression levels of genes associated with trilineage differentiation relative to levels of expression in EBV-iPSCs, 6 factors-T-iPSCs exhibited upregulation of mesodermal differentiation capacity relative to that in Con-T-iPSCs (Figure 2D). We also compared the trilineage differentiation capacities of EBV-iPSCs, Con-T-iPSCs, and 6 factors-T-iPSCs with those of 13 undifferentiated standard hPSCs programmed in the hPSC Scorecard algorithm,^21^ which also displayed relative upregulation of mesodermal differentiation capacity, important for hematopoiesis, in 6 factors-T-iPSCs (Figure 2E).

**Figure 3:**
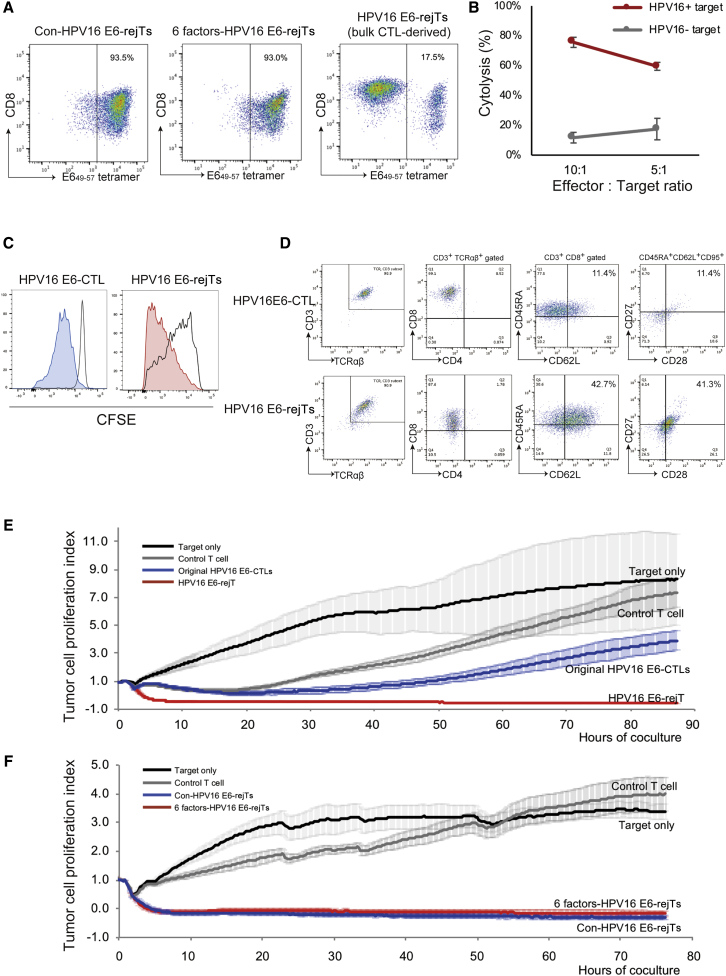
Anti-Cervical Cancer Effect of HPV16 E6-rejTs as Examined *In Vitro* (A) Flow cytometric E6_49–57_ tetramer analysis of Con-HPV-rejTs, 6 factors-HPV-rejTs, and bulk CTL-derived HPV-rejTs. (B) *In vitro*^51^Cr-release assay with HPV16 E6-rejTs (effector) and HLA-matched HPV16^+^ cervical cancer cell line SKG-IIIa (target). HLA-matched HPV16^−^ tumor cell line, control target. The effector-to-target (E:T) ratios were 10:1 and 5:1. The mean percentages of specific tumor cell lysis ± SD are shown. (C) CFSE dilution T cell proliferation assay, HPV-CTLs and HPV-rejTs. (D) Representative flow cytometric analysis of T cell subset and memory phenotype (CD45RA and CD62L population) of original HPV16 E6-CTLs and of HPV16 E6-rejTs. (E) RTCA continuous graphical output of tumor proliferation index up to 85 h for SKG-IIIa cocultured with original HPV16 E6-CTLs, 6 factors-HPV16 E6-rejTs, and control rejTs; also tumor. E:T ratios were uniformly 1:1. HLA-mismatched different epitope-rejTs, control. The data shown represent at least three independent triplicate experiments. Mean values were plotted ±SD. (F) RTCA continuous graphical output of tumor proliferation index up to 75 h for SKG-IIIa cocultured with 6 factors-HPV16 E6-rejTs, Con-HPV16 E6-rejTs, and control rejTs; also tumor only. E:T ratios were uniformly 1:1. HLA-mismatched different epitope-rejTs, control. The data shown represent at least three independent triplicate experiments. Mean values were plotted ±SD.

We finally differentiated T-iPSCs into functionally rejuvenated CTLs in accordance with our established T cell redifferentiation protocol.^17^, ^18^, ^19^, ^20^ HPV16 E6-rejTs were efficiently redifferentiated from both Con-T-iPSCs and 6 factors-T-iPSCs. Con-T-iPSC-derived HPV-rejTs (Con-HPV-rejTs) and 6 factors-T-iPSC-derived HPV-rejTs (6 factors-HPV-rejTs) maintained the same antigen specificity as that of original CTLs (93.5% and 93.0% for tetramer positivity, respectively) (Figure 3A, left and center). In contrast, the E6_49–57_ tetramer^+^ fraction of HPV-rejTs derived from T-iPSCs that were established from bulk HPV-CTLs was low (only 17.5% for tetramer positivity) (Figure 3A, right).

This might result from inclusion of a very few non-specific CTLs among purified bulk HPV-CTLs (Figure 1D). The small population of miscellaneous CTLs can engender a large population of non-specific rejTs after reprogramming and differentiation. These findings suggest that 6 factors-T-iPSCs differentiated into HPV16 E6-rejTs as efficiently as did Con-T-iPSCs and that single-cell cloning is important for maintenance of high antigen specificity of rejTs after redifferentiation.

### iPSC-Derived HPV16 E6-rejTs Exhibited More Robust and Longer Sustained Cytotoxicity Than Did Original HPV-CTLs

To evaluate antigen-specific antitumor effects of HPV16 E6-rejTs, we performed 6-h coculture chromium-51 (^51^Cr) release assays and compared the percentage cytolysis of HPV-rejTs in the HLA-matched HPV16^+^ cervical cancer cell line SKG-IIIa with that in of HPV16^−^ control target cells. The percentages of HPV16^+^ target cells lysed by HPV-rejTs were 75.6% and 59.5% (effector-to-target [E:T] ratios of 10:1 and 5:1, respectively), whereas those of HPV16^−^ control target cells lysed by HPV-rejTs were 11.7% and 17.5% (E:T ratios of 10:1 and 5:1, respectively) (Figure 3B). We also compared the proliferating capacities of HPV16 E6-rejTs and of the original HPV16 E6-CTL clone by carboxyfluorescein succinimidyl ester (CFSE) dilution T cell proliferation assays after T cell receptor stimulation. The proportion of proliferating cells among HPV16 E6-rejTs was greater than that among HPV16 E6-CTLs on day 7 after stimulation (72.2% versus 40.9%), showing that HPV16 E6-rejTs proliferated more vigorously than did the original HPV-CTL clone (Figure 3C). To compare the phenotype of HPV16 E6-rejTs with that of the original HPV16 E6-CTL clone, surface molecules relating to their lineage subtype and memory phenotype were evaluated by flow cytometry. HPV-rejTs and CTL clone expressed CD3, TCRαβ, and CD8. HPV-rejTs were CD45RA^+^, CD62L^+^, CD95^+^, CD27^+^, and CD28^+^, representing stem cell memory phenotype.^22^^,^^23^ Alternatively, original HPV16-CTLs contained many effector T cells and a small number of cells with stem cell memory phenotype (CD45RA^+^, CD62L^+^: rejTs 42.7% versus CTLs 11.4%; CD95^+^, CD27^+^, CD28^+^: rejTs 41.3% versus CTLs 11.4%) (Figure 3D).

We further compared antitumor activity of HPV-rejTs and peripheral blood-derived original HPV-CTLs against cervical cancer using real-time cell analysis (RTCA). SKG-IIIa cells continuously proliferated when T cells were not added in coculture, whereas coculture with HPV16 E6-rejTs robustly suppressed SKG-IIIa cell proliferation. Original HPV16 E6-CTLs also suppressed SKG-IIIa cell proliferation, but not so persistently as did HPV-rejTs. At 5 h of coculture, HPV16 E6-rejTs exhibited greater killing effects than did original HPV16 E6-CTLs (86.6% killed versus 34.1%, p < 0.0001). The percentage of HPV16 E6-rejT killing reached >90% within 10 h of coculture, a percentage maintained until experiments ended (85 h). In contrast, original HPV16 E6-CTLs started to kill SKG-IIIa cells gradually and reached their strongest killing effects (82.4%) 30 h after coculture began. However, tumor growth later surpassed antitumor activity. At 85 h, the difference of percentage killing between HPV16 E6-rejTs and HPV16 E6-CTLs became evident (96.6% versus 42.4%, p = 0.0007) (Figure 3E).

We also compared anti-tumor activity of Con-HPV-rejTs with that of 6 factors-HPV-rejTs using coculture and RTCA. At no time did cytotoxicity against HPV16^+^ cervical cancer cells differ significantly between Con-HPV-rejTs and 6 factors-HPV-rejTs. Both types of HPV16 E6-rejTs completely suppressed tumor growth within the first 10 h of coculture; the anti-tumor effects persisted for more than 75 h (Figure 3F). Collectively, stem cell memory phenotype HPV16 E6-rejTs exhibited antigen-specific, rapid, and sustained anti-cervical cancer activity, with no significant difference in anti-tumor effect between Con-HPV-rejTs and 6 factors-HPV-rejTs.

### HPV16 E7-rejTs Also Showed More Robust Cytotoxicity Than Did Original HPV16 E7-CTLs

To examine the cytotoxicity of HPV16 E7-specific rejTs against cervical cancer, T-iPSCs established from bulk HPV16 E7-CTLs were redifferentiated into HPV16 E7-rejTs. We picked up multiple T-iPSC single colonies and differentiated five randomly selected T-iPSC lines into five lines of HPV16 E7-rejTs.

**Figure 4:**
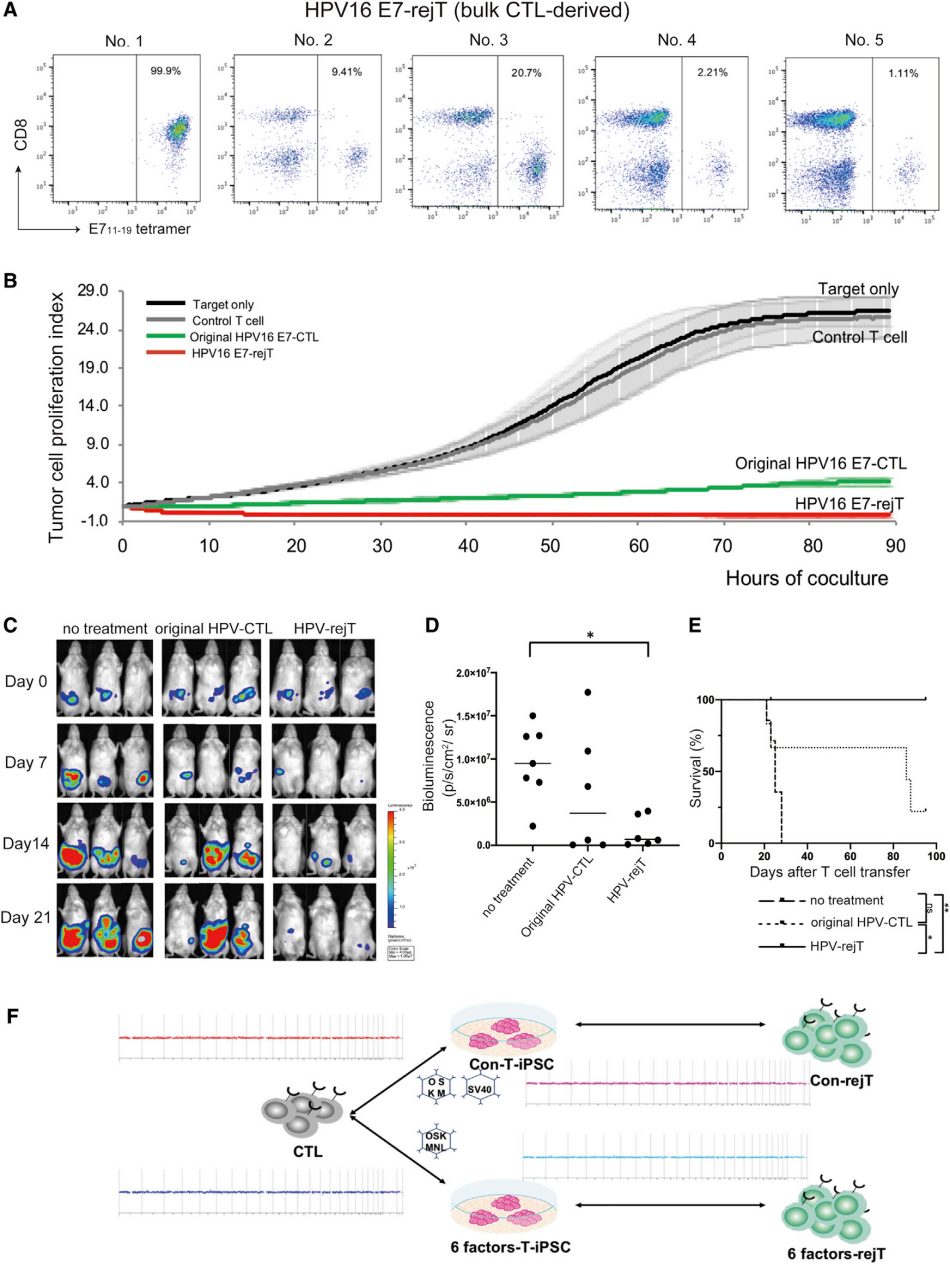
Anti-Cervical Cancer Effect of HPV16 E7-rejTs as Examined *In Vitro* and Sustainable Tumor-Suppressive Effect of HPV16 E6-rejTs *In Vivo* (A) Flow cytometric E7_11–19_ tetramer analysis of each line of bulk CTL-derived HPV-rejTs. (B) RTCA continuous graphical output of tumor proliferation index up to 85 h for CaSki cells cocultured with HPV16 E7-rejTs, original bulk HPV16 E7-CTLs, and control CTLs; also tumor only. HLA-mismatched different epitope-CTLs, control. E:T ratios were uniformly 1:1. Data were plotted and are shown as mean ± SD. (C) Bioluminescence imaging of mice treated with original HPV16 E6-CTL clone or HPV16 E6-rejTs. *GFP/FFluc*-labeled cervical cancer-bearing mice were divided into three groups that received no treatment (n = 7), HPV-CTLs (n = 6), or HPV-rejTs (n = 6). Images of three representative mice from each group are shown. (D) Quantification of tumor burden on day 21 is represented. ∗p < 0.05 by one-way ANOVA. (E) Kaplan-Meier survival curves representing percentage survival of the experimental groups: no treatment, HPV-CTLs, or HPV-rejTs. ∗p < 0.05, ∗∗p < 0.01 and by log-rank testing. ns, not significant. (F) Results of comparative genomic hybridization microarray of HPV-CTLs, Con-T-iPSCs, Con-HPV-rejTs, 6 factors-T-iPSCs, and 6 factors-HPV-rejTs. No aberrations in copy numbers in all comparisons: original HPV-CTLs versus Con-T-iPSCs, Con-T-iPSCs versus Con-HPV-rejTs, original HPV-CTLs versus 6 factors-T-iPSCs, and 6 factors-T-iPSCs versus 6 factors-HPV-rejTs.

Positivity for E7_11–19_ tetramer varied from 1.11% to 99.9% (Figure 4A). E7 antigen specificity in HPV16 E7-rejTs originating from bulk HPV16 E7-CTLs was low in all lines but one.

Both HPV16 E7-rejTs and original HPV16 E7-CTLs effectively suppressed CaSki cell proliferation, but the cytotoxicity of HPV16 E7-rejTs was stronger than that of original HPV16 E7-CTLs on RTCA (96.8% versus 81.6%, p = 0.0218, 85 h) (Figure 4B). HPV16 E7-rejTs thus were demonstrated to exert robust cytotoxicity against cervical cancer. However, although HPV16 E7-rejTs with high antigen specificity could be generated from purified bulk HPV16 E7-CTLs, to pick up many T-iPSC colonies and differentiate them into many rejT lines was laborious and inefficient.

### Robust Tumor-Suppressive Effect and Survival Advantage of HPV16 E6-rejTs Were Demonstrated *In Vivo*

To elucidate whether HPV-rejTs also showed a strong anti-tumor effect *in vivo*, SiHa cells (an HPV16^+^ cervical cancer cell line) transduced with retrovirus-derived firefly luciferase (1 × 10^5^ cells/mouse) were intraperitoneally engrafted into non-obese diabetic (NOD)/Shi-severe combined immunodeficiency (scid)interleukin(IL)2Rγnull (NOG) mice. Light emission was monitored as an indicator of tumor growth. Once bioluminescence occurred and progressively increased (3 d after tumor inoculation), mice were divided into three groups. No treatment was given in one control group (n = 7), and HPV16 E6-CTLs (n = 6) or HPV16 E6-rejTs (n = 6) were injected in the two treatment groups (2.5 × 10^6^ per dose, three doses) followed by intraperitoneal injection of recombinant human interleukin (IL)-2, 1,000 U/mouse, five times a week. Bioluminescence rapidly increased in mice without treatment over time. In contrast, HPV-rejTs effectively suppressed tumor growth, and HPV-CTLs also tended to suppress tumor proliferation by 21 d after T cell transfer, with clearer anti-tumor effects having been found with HPV-rejT treatment (Figure 4C). Compared with untreated mice, HPV-rejTs produced a significant reduction in tumor burden on day 21 (p = 0.0249) (Figure 4D). On long-term observation, HPV-rejTs significantly prolonged survival beyond that in mice receiving original HPV-CTLs (p = 0.0309) and receiving no treatment (p = 0.0073) (Figure 4E). Taken together, these results suggest that HPV16 E6-rejTs robustly suppressed cervical cancer and markedly prolonged survival *in vivo*.

### No Chromosomal Abnormalities Were Found by Comparative Genomic Hybridization Microarray in Either Con-HPV-rejTs or 6 Factors-rejTs

To evaluate tumorigenesis risk of the process in generation of HPV-rejTs, we performed comparative genomic hybridization microarray assays. No deletions, amplifications, breakpoints, or ploidy abnormalities of oncogenes/tumor-suppressor genes were detected on any comparison (Figure 4F); these included original HPV16 E6-CTL clone versus Con-T-iPSCs, Con-T-iPSCs versus Con-HPV-rejTs, original HPV16 E6-CTL clone versus 6 factors-T-iPSCs, and 6 factors-T-iPSCs versus 6 factors-HPV-rejTs. Neither establishment of T-iPSCs using Con-SeV or 6 factors-SeV nor differentiation from T-iPSCs into HPV-rejTs caused genomic abnormalities.

## Discussion

With any kind of iPSC-derived cells, to achieve the goal of clinical use risks associated with reprogramming must be reduced. However, to enhance efficiency in reprogramming original CTL single-cell clones, cotransduction of SV40 large T antigen has been necessary.^19^^,^^24^^,^^25^ SV40 large T antigen inactivates p53 tumor-suppressor protein,^26^ causing genomic instabilities and tumorigenesis.^27^ For clinical use, the avoidance of SV40 large T antigen thus is preferable, even though interaction of SV40 large T antigen with the genome is temporary. We could establish T-iPSCs from CTL single-cell clones using 6 factors-encoded SeV without SV40 large T antigen. These SeV vectors encode microRNA to eliminate SeV.^28^ Thus, after SeV has expressed exogenous reprogramming factors, it rapidly disappears from the reprogrammed cells. This is important for avoiding undesired side effects such as cell transformation and insertional mutagenesis.^29^ Also noteworthy is that incorporation of six reprogramming factors into a single SeV vector means that all reprogramming factors can be simultaneously expressed at a constant ratio in a single cell,^29^ whereas other types of SeV have usually required multiple vectors to express all reprogramming genes.^29^, ^30^, ^31^ When each of the reprogramming genes is installed via separate vectors, of concern is that their expression levels are likely to vary among transduced cells, which might affect the quality of T-iPSCs.^29^ T-iPSC establishment reprogrammed by a single 6 factors-SeV vector therefore is thought better than conventional approaches. Moreover, HPV-rejTs efficiently differentiated from 6 factors-T-iPSCs exhibited 100% tetramer positivity and strong cytotoxicity against cervical cancer. The antitumor activity of HPV-rejTs was much stronger than that of HPV-CTLs. These findings may reflect robust proliferating capacity and persistence of rejTs.^17^, ^18^, ^19^, ^20^ Long-term cytotoxicity can show trends such as that of *in vivo* antitumor activity.^32^ Our *in vivo* results actually demonstrated that, compared with HPV-CTLs, HPV-rejTs distinctly suppressed cervical cancer proliferation and showed a survival advantage. HPV16 E6- and HPV16 E7-rejTs thus may be a promising therapy for cervical cancer.

We also showed that in establishing T-iPSC single-cell cloning is essential to maintain the desired T cell receptor (TCR) in the rejTs. When we omitted single-cell cloning, more non-specific rejTs arose and antitumor activity per effector cell was lower. Furthermore, non-specific rejTs that have various TCRs may cause autoimmune reactions such as acute graft-versus-host disease. These findings established for us that single-cell cloning is quite important, even though it takes much time.

Of interest is that HPV16 E6- and HPV16 E7-specific CTLs could be obtained from peripheral blood mononuclear cells (PBMCs) of healthy donors but not from those of two heavily treated cervical cancer patients. This suggests a limitation of autologous cell resources for cervical cancer patients, as CTLs of cervical cancer patients are thought to be seriously exhausted.

Banked HLA-matched or HLA-edited allogeneic cell resources from healthy donors will be better suited to clinical use in this context. If healthy donors had been previously exposed to HPV16, HPV16-CTLs could be generated. Another possibility is that if the donor had been exposed to cross-reactive antigens, we could possibly generate HPV16-CTLs. The development of HLA class I-edited universal cells to prevent immunorejection may be a realistic solution. HPV-rejT therapy against refractory cervical cancer utilizing iPSC technology offers us access to stable and abundant cell resources that permit establishment of “off-the-shelf” T cell therapy.

In conclusion, this study demonstrated that use of 6 factors-SeV enabled establishment of T-iPSCs as an unlimited source of HPV16-rejTs in a manner safer than conventional approaches and that generated HPV16-rejTs thus exhibit a more robust and sustained anti-tumor effect against cervical cancer than do original peripheral blood-derived HPV-CTLs not only *in vitro* but also *in vivo*. These findings support clinical use of HPV16 E6- and E7-rejTs against cervical cancer.

## Materials and Methods

### Generation of HPV16-Specific CTLs

We recruited donors through the Department of Obstetrics and Gynecology, Juntendo University School of Medicine. This study was conducted in accordance with the Declaration of Helsinki and was approved by the Ethics Committee at Juntendo University School of Medicine. Peripheral blood from patients or healthy donors bearing HLA-A^∗^2402 or HLA-A^∗^0201 was obtained with informed consent. Healthy donors were confirmed as HPV16-seropositive by immunochromatography.^33^ Briefly, for generation of HPV16-specific CTLs we cocultured PBMCs with dendritic cells loaded with A∗2402/HPV16 E6_49–57_ or A∗0201/E7_11–20_ custom-synthesized peptides (Mimotopes, Mulgrave, VIC, Australia) in the presence of 400 U/mL of IL-4 and 10 ng/mL of IL-7 (both Miltenyi Biotec, Bergisch Gladbach, Germany) to increase the frequencies of virus antigen-specific CTLs.^34^^,^^35^ On day 16, T cells were harvested and antigen specificity against HPV16 E6 and HPV16 E7 was determined by staining with HLA-A∗2402/E6_49–57_ tetramer and HLA-∗0201/E7_11–19_ tetramer, respectively (MBL International, Nagoya, Japan). The cells to which tetramer bound were single-cell cloned by limiting dilution or bulk cultured after tetramer-phycoerythrin (PE)-coupled/anti-PE-microbeads magnetic cell separation (Miltenyi Biotec).^17^, ^18^, ^19^, ^20^

### Reprogramming HPV-CTLs into T-iPSCs

We attempted reprogramming of HPV-CTL clones by inserting two types of reprogramming factor sets into T-iPSCs. One was the conventional reprogramming factor set for CTL clones, using two SeV vectors loaded with the OSKM and SV40 large T antigen to improve reprogramming efficiency (Con-SeV).^17^, ^18^, ^19^, ^20^ The other was 6 factors-SeV loaded with six factors (OSKM plus NANOG and LIN28). HPV16-E6 CTL clones were transduced with SeV at a multiplicity of infection of 10. Transduced cells were seeded onto six-well plates coated with recombinant laminin, iMatrix-511 (Nippi, Tokyo, Japan). Seeded cells were cultured in CTL medium in the presence of 100 U/mL of IL-2 (Miltenyi Biotec), and the medium was replaced with StemFit AK03N (Ajinomoto Healthy Supply, Tokyo, Japan).

### Immunocytochemical Staining

T-iPSCs were incubated with a primary antibody against SeV NP (mouse monoclonal, clone #2E4, 1:1,000)^36^ after fixation with 4% paraformaldehyde,^28^ followed by staining with goat anti-mouse immunoglobulin G (IgG) conjugated with Alexa Fluor 488 (1:500, Invitrogen). Nuclei were counterstained with 4′,6-diamidino-2-phenylindole (DAPI, 1:1,000, Roche Diagnostics, Mannheim, Germany). Photomicrographs were obtained using a BZ-X800 fluorescence microscope (Keyence, Osaka, Japan).

### Quantitative Real-Time PCR

Total RNA was extracted from established T-iPSCs using TRIzol (Invitrogen), and cDNA was synthesized using a ReverTra Ace qPCR RT kit (Toyobo, Osaka, Japan). Target SeV and PCR primer sequences used to quantitate residual SeV vectors by quantitative real-time PCR are shown in Table 1. Gene expression levels associated with self-renewal and trilineage differentiation in established T-iPSCs were characterized as well, using the TaqMan hPSC Scorecard panel (Life Technologies, Carlsbad, CA, USA). We used EBV-iPSCs as control T-iPSCs (confirmed as pluripotent by teratoma formation). All quantitative real-time PCR procedures were carried out on the StepOnePlus real-time PCR system (Applied Biosystems, Carlsbad, CA, USA).^37^ Individual PCR reactions were normalized against GAPDH levels.

**Table 1:** PCR Primers and a Probe for Detecting Residual SeV Vector

Gene		Primer
6 Factors-SeV	NP	5′-GGAAGGAATCGGCTCAGTGATG-3′
		5′-GGGCCGTGTTCATGGTCAC-3′
Gene		Probe
6 Factors-SeV	NP	5′-FAM-AGAGTTTCCACCATCAGCGACACC AGGG-QSY-3′

PCR, polymerase chain reaction; SeV, Sendai virus; NP, nucleocapsid protein.

### Differentiation of T-iPSCs into HPV16 E6- and E7-Specific Rejuvenated CTLs

Using microscopy, we manually picked up each T-iPSC colony when stable colonies were found. T-iPSCs were differentiated into HPV16 E6- and E7-specific rejTs as described.^17^, ^18^, ^19^, ^20^ Briefly, small clumps of T-iPSCs were transferred onto C3H10T1/2 cells with coculture in Iscove’s modified Dulbecco’s medium (Sigma-Aldrich) supplemented with 15% fetal bovine serum (FBS) (HyClone, GE Healthcare, South Logan, UT, USA) and a cocktail of 10 mg/mL human insulin, 5.5 mg/mL human transferrin, 5 ng/mL sodium selenite, 2 mM l-glutamine (Thermo Fisher Scientific), 0.45 mM α-monothioglycerol (Wako Pure Chemicals, Osaka, Japan), and 50 mg/mL ascorbic acid (Takeda, Osaka, Japan) in the presence of 20 ng/mL vascular endothelial growth factor (Miltenyi Biotec). Hematopoietic cells collected from iPSC sac contents were transferred onto DL1/4-expressing C3H10T1/2 feeder cells for T-lineage differentiation during coculture in α-minimum essential medium (α-MEM) (Thermo Fisher Scientific) supplemented with 20% FBS (HyClone, GE Healthcare), as well as penicillin-streptomycin-glutamine in the presence of 20 ng/mL recombinant human stem cell factor (Miltenyi Biotec), 10 ng/mL Fms-related tyrosine kinase 3 ligand (Miltenyi Biotec), and 10 ng/mL IL-7. T-lineage cells were harvested, stimulated with 5 mg/mL phytohemagglutinin-L, mixed with irradiated PBMCs, and cocultured in CTL medium in the presence of 10 ng/mL IL-7 and 10 ng/mL IL-15. Both C3H10T1/2- and DL1/4-expressing C3H10T1/2 feeder cell lines are compatible with good manufacturing practice standards for clinical use. Master cell bank stocks were made.

### Antibodies

Allophycocyanin (APC)/cyanin 7 (Cy7)-conjugated mouse anti-human CD3, APC-conjugated mouse anti-human CD4, Pacific Blue-conjugated mouse anti-human CD8α, Alexa Fluor 488-conjugated mouse anti-human TCRαβ, Pacific Blue-conjugated mouse anti-human CD45RA, PE-conjugated mouse anti-human CD62L, APC/Cy7-conjugated mouse anti-human CD95 (all BioLegend, San Diego, CA, USA), V500-conjugated mouse anti-human CD8, Alexa Fluor 700-conjugated mouse anti-human CD3, APC-conjugated mouse anti-human CD27, and fluorescein isothiocyanate-conjugated mouse anti-human CD28 (all BD Biosciences, San Jose, CA, USA) were used to label cells for flow cytometry analysis.

### Flow cytometry

Flow cytometry analysis was carried out on BD FACSAria II or BD LSRFortessa equipment (BD Biosciences) and the acquired data were analyzed with FlowJo software 10.5.3 (Tree Star, Ashland, OR, USA). Propidium iodide was used to gate in live cells in all analyses. A fluorescence-minus-one technique was used to interpret flow cytometry data in all antibody combinations.

### Tumor Cell Lines

The HPV16^+^ cervical cancer cell lines SKG-IIIa (HLA-A24^+^), CaSki (HLA-A02^+^) (both RIKEN BioResource Research Center, Tsukuba, Ibaragi, Japan), and SiHa (HLA-A24^+^) (ATCC, Manassas, VA, USA) were cultured in RPMI 1640 medium (Sigma-Aldrich) supplemented with 10% FBS and penicillin-streptomycin-glutamine (Thermo Fisher Scientific). The HPV16^−^ malignant lymphoma cell line NK-YS (HLA-A24^+^) (kindly provided by Dr. Junjiroh Tsuchiyama, Okayama University Medical School, Okayama, Japan) was grown in Iscove’s modified Dulbecco’s medium supplemented with 10% FBS and 100 U/ml of IL-2.^38^

### ^51^Cr-Release Assay

The cytotoxicity of HPV-rejTs against the HPV16^+^ cervical cancer cell line (SKG-IIIa) and against an HPV16^−^ control cell line (NK-YS) was measured by a ^51^Cr-release assay at E:T ratios of 10:1 and 5:1 for each cell type. Target cells were labeled with ^51^Cr for 1 h and HPV-rejTs were cocultured with target cells for 6 h. Percentages of cytolysis were calculated as ([experimental release − spontaneous release]/[maximum release − spontaneous release]) × 100 (%).

### T Cell Proliferation Assay

HPV-CTLs (1 × 10^6^) and HPV-rejTs (1 × 10^6^) were labeled with 1 μM CFSE (Invitrogen) and stimulated with T Cell TransACT (Miltenyi Biotec) following the manufacturer’s protocols. CFSE is equally distributed between daughter cells upon mitosis, resulting in loss of signal by flow cytometry, and thus is useful in tracking cell divisions. In this study, the labeled stimulated cells were analyzed by flow cytometry on days 3 and 7 after stimulation. CFSE fluorescence was collected in the fluorescein isothiocyanate channel by flow cytometry.

### Impedance-Based Tumor Killing Assay

RTCAs (xCELLigence, ACEA Biosciences, San Diego, CA, USA) were performed to verify tumor-suppressive effects for both original HPV-CTLs and HPV-rejTs. In each experiment, we cocultured an HPV16^+^ cervical cancer cell line with original HPV-CTLs, HPV-rejTs, and epitope-mismatched rejTs; the last served as a control effector. Briefly, target cells (1 × 10^4^ SKG-IIIa or CaSki cells per well) were seeded and cultured for 8–24 h in microelectrode-coated 96-well plates to permit target cell adherence. Effector cells were then added (E:T ratio, 1:1). Electrical impedance changes were recorded automatically and continuously as cell index (CI) (Figures 3E, 3F, and 4B). For each plot, the y axis indicates CI and x axis indicates time in hours. Triton X-100 (0.2%) was added to some wells to permit definition of a complete cell lysis index (CI_max_). All RTCAs were carried out in triplicate in at least three independent assays to ensure reproducibility. Percentages of killing (% killing) were calculated as ([CI_no effector_ − CI_effector_]/[CI_no effector_ − CI_max_]) × 100 (%).^13^^,^^32^

### Antitumor Activity in *In Vivo* Model

All *in vivo* studies were approved by the Animal Research Committees of Juntendo University School of Medicine. To verify the tumor-suppressive effect of HPV16 E6-CTLs and HPV16 E6-rejTs against HPV16^+^ cervical cancer, a cervical cancer cell line, SiHa, was transduced with a γ-retroviral vector encoding the fusion protein *GFP/FFluc*. Six-week-old female NOG mice (In-Vivo Science, Tokyo, Japan) were engrafted intraperitoneally with *GFP/FFluc* SiHa (1 × 10^5^ cells/mouse) and tumor growth was monitored using the Caliper *in vivo* imaging system (IVIS) imaging system (Caliper Life Sciences, Mountain View, CA, USA). Firefly d-luciferin substrate (OZ Biosciences, Marseille, France) was intraperitoneally injected into mice 15 min before imaging. Mice were divided into three groups, two treatment groups and one no treatment group. Three days after tumor inoculation, mice were treated intraperitoneally with original HPV-CTLs or with HPV16 E6-rejTs (2.5 × 10^6^ cells, three doses, once a week). Living Image software version 4.7.2 (PerkinElmer) were used for luminescence analyses. The intensity of signal was measured as total photon/s/cm^2^/steradian (p/s/cm^2^/sr) as described^20^^,^^39^.

### Analyses of Oncogenes and Tumor Suppressor Genes by Comparative Genomic Hybridization Microarray

To assess tumorigenesis within the cells generated, oncogenes and tumor suppressor genes of original peripheral blood-derived HPV-CTLs, T-iPSCs, and iPSC-derived HPV-rejTs were examined by comparative genomic hybridization microarray.^40^ The 12,055 probe contents were selected from 1,675 oncogenes and tumor suppressor genes (Agilent SurePrint G3 human comparative genomic hybridization (CGH) microarray 8×60K; Agilent Technologies, Santa Clara, CA). Sample labeling and array hybridization were performed according to a two-color microarray-based protocol (Agilent Technologies). After hybridization, the scanned TIFF images were processed by Feature Extraction 11.5.1.1 software (Agilent Technologies). Data were analyzed by Agilent Genomics WorkBench software using the Aberration Detection Method 2 algorithm (Agilent Technologies).

### Statistical Analysis

All data are presented as mean ± SD or SEM. Results were analyzed by an unpaired Student’s t test (two-tailed) or ANOVA as stated, with a p value <0.05 indicating a significant difference. Survival curves were compared using Kaplan-Meier analysis with log-rank testing. Software for all statistical analyses was Excel (Microsoft, Redmond, WA, USA) and Prism 8.0 (GraphPad Software, San Diego, CA).

## Author Contributions

M.A. planned and performed the experiments and wrote the manuscript. T.H. performed the experiments and wrote the manuscript. Y.S. and M.I. helped in CTL generation. J.A. helped in performing ^51^Cr-release assays. K.O. helped in iPSC establishment and iPSC culture. M.O. and M.N. provided SeV vector. M.I. and T.T. helped in animal experiments and in performing quantitative real-time PCR. Y.T., A.M., and J.A. recruited patient and healthy donors. H.N. directed the study and wrote the manuscript. N.K. provided scientific discussions.

## Conflicts of Interest

H.N. is a co-founder of and an advisor to Century Therapeutics. The remaining authors declare no competing interests.
